# Utility of FDG PET/CT in Sjögren’s Syndrome and associated lymphomas; Lymphomagenesis

**DOI:** 10.22038/aojnmb.2024.76893.1541

**Published:** 2025

**Authors:** Dikhra Khan, Prateek Kaushik, Sambit Sagar, Jasim Jaleel

**Affiliations:** 1 India Department Of Nuclear Medicine All India Institute of Medical Sciences, Delhi, India; 2 Department of Nuclear Medicine, Institute of Liver and Biliary Sciences, New Delhi, Delhi, India

**Keywords:** Primary Sjögren syndrome, Lymphoma, Lymphomagenesis, FDG, PET/CT

## Abstract

Primary Sjögren syndrome (SS) is an autoimmune disease affecting exocrine glands, with predisposition to development of lymphoma (lymphomagenesis). We report a case of Sjogren’s syndrome and discuss the role of FDG PET/CT in the primary diagnosis of lymphoma transformation in SS. Furthermore, we reviewed the literature regarding the utility of FDG PET/CT to assess systemic disease activity and also its role in the SS associated lymphoma with light into the new PET tracers that can be explored for these indications in the future. Published data suggest promising role of FDG PET/CT in SS associated lymphomas, and demands larger studies for its establishment.

## Introduction

 Primary Sjögren’s syndrome (SS) is a systemic disorder with features of lymphocytic infiltration and subsequently leading to progressive destruction of the exocrine glands (1). Due to its systemic manifestations of the ongoing inflammatory process, it can present with a broad spectrum of signs and symptoms involving any organs beyond the exocrine glands, thereby leading to dryness, pain, and fatigue(2). Nearly 30–50% of patients with Sjogren’s syndrome presents with severe systemic manifestations (3, 4). Nevertheless, dryness of mouth and eyes, remain the most common characteristic manifestation of SS. It has been previously reported that imaging techniques can assist in the challenging diagnostic process of SS by assessing disease activity and determining disease progression (5). ESSDAI (EULAR Sjögren's Syndrome Disease Activity Index) score has been described previously to help recognize patients with active disease, however particular tools to better determine underlying immunological activity in SS are still lacking (6). Furthermore, we reviewed the literature regarding role of FDG PET/CT in Sjögren syndrome on MEDLINE, EMBASE and SCOPUS until March 2023. Key terms used in the research were “FDG PET/CT” “Sjögren syndrome” and “Lymphoma”. We selected eight articles (12-14, 16, 19, 20, 29, 30) that reported transformation of Sjögren syndrome to lymphoma. There was no language restriction, and we included all reports, excluding studies reported only as abstracts.

## Case Report

 A 41-year-old female with Sjogren’s syndrome since 2015 presented with substantial swelling of the eyes and parotid gland four months ago. 

 She was treated with steroid and DMARD for 5 years. A general examination revealed palpable bilateral cervical lymph nodes. Other systematic examinations did not show any remarkable findings. MRI sialography was suggestive of bilateral parotiditis.

 In detail her hematological, biochemistry and serological profile was as given in the tables with other serologic and immunologic investigations being negative.

**Table:** 

**Serologic and Immunologic Investigations**	**Results**
HBV	Negative
HCV	Negative
HIV	Negative
EBV	Negative

**Figure 1:**
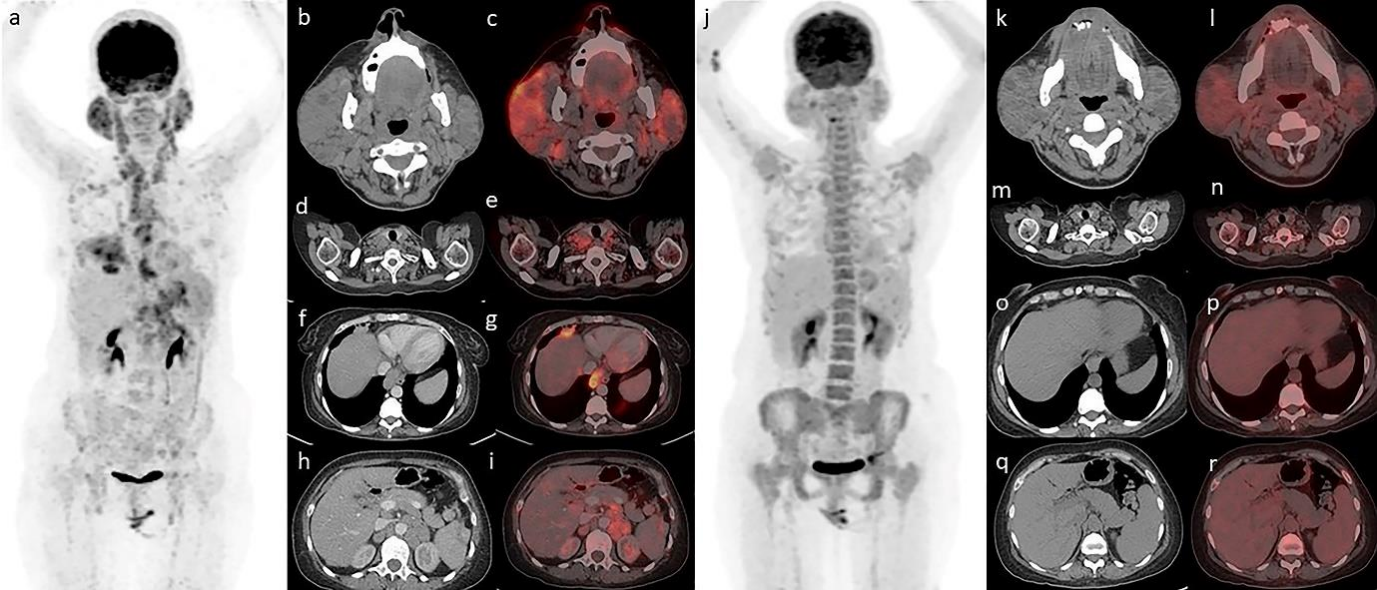
FDG PET/CT showed metabolically active enlarged bilateral parotid (**b**,**c**) and lacrimal glands with cervical, supraclavicular (**d**,**e**), mediastinal, anterior diaphragmatic (**f**,**g**), and abdomino-pelvic lymph nodes (**h**,**i**) along with hepatosplenomegaly. Compared to previous PET/CT scan (**j**, **k**, **r**) there was an increase in size and uptake of the bilateral parotid glands along with the new lesions

 With the suspicion of a high risk of development of lymphoma in Sjogren’s syndrome, she underwent FDG PET/CT (Figure 1). The FDG PET/CT showed metabolically active enlarged bilateral parotid and lacrimal glands (Figure 1 b,c ) with cervical, supraclavicular (Figure 1 d,e), mediastinal, anterior diaphragmatic (Figure 1 f,g), and abdomino-pelvic lymph nodes (Figure 1 h,i) along with hepatosplenomegaly. This patient has previously also undergone FDG PET/CT (Figure 1 j) in our department 10 months back, in suspicion of development of Lymphoma. 

 Compared to previous PET/CT scan (SUV_max_=4.2) there was increase in size and uptake of the bilateral parotid glands (SUV_max_=7.9) along with new appearance of all the above-mentioned lesions. These findings were highly suggestive of lymphomagenesis and we suggested biopsy from the right parotid gland (in view of increased size and high metabolic uptake). Histopathology examination from the right parotid swelling revealed MALT (Mucosa-associated lymphoid tissue) lymphoma with immuno-histochemical markers positive for CD 20 and negative for CD30 & CD136.

## Discussion

 Sjögren’s syndrome is a chronic systemic autoimmune disease that predisposes to the development of lymphoproliferative disorders of B cell origin. It is associated with the highest risk of lymphoma, particularly non-Hodgkin’s lymphoma, with a prevalence in the range from 2.7% to 9.8% (7). This process of lymphomagenesis is a multistep process that is genetically contributed by the interaction of SS-related BAFF gene haplotypes (30). The most common histologic type is marginal-zone lymphoma, particularly of the mucosa-associated lymphoid tissue (MALT) type. Nearly 65% of SS patients with associated lymphomas suffer from type of indolent extranodal marginal zone B cell lymphoma. The other 2 less common histologic types include diffuse large B cell lymphomas (15%) and nodal marginal zone B cell lymphomas (10%). The clinical manifestations most commonly noted in lymphoma associated patients were parotidomegaly (67%) and lymphadenopathy (61%) and also 70% of patients present with hematologic manifestations including anemia, neutropenia or thrombocytopenia. It has been previously reported that the median time from SS to lymphoma diagnosis is around 7.5 years (8-11). The diagnosis of lymphoma is sometimes challenging, notably as clinical symptoms usually observed in lymphoma, such as lymphadenopathy are commonly detected in case of benign B cell activation in lieu of absence of malignant clonal proliferation (4). In addition, these lymphomas are low grade specifically involving extranodal sites (i.e., the salivary glands), and also they are not usually associated with major constitutional symptoms or hematologic abnormalities. MALT lymphomas are known to arise commonly within the parotid glands, however it has been reported to develop at other extranodal locations, such as the lungs, lacrimal glands, or stomach (12-14). EULAR Sjögren's syndrome disease activity index (ESSDAI) is a systemic disease activity index that was outlined to measure disease activity in patients with primary SS. It can assist in identification of patients with active disease (15, 16). In 2013, Cohen et al reported that a new PET/CT activity score, based on ^18^F-FDG PET/CT (^18^F-labeled fluorodeoxyglucose–positron emission tomography) can correlate with ESSDAI and may help to assess disease activity in SS patients (16). In SS patients, several case reports depicted abnormal FDG uptake in salivary glands however, physiological FDG uptake in salivary glands is highly variable, which possibly may be due to varied glucose utilization for metabolism in different subjects (20-25).

 Multiple studies have reported the role of FDG PET/CT in detecting salivary gland inflammation, along with systemic disease activity (17-21). Even though salivary gland inflammation can be shown by FDG PET/CT. 

 Several authors also reported its ability to detect systemic disease activity that is visualized, mainly within salivary glands, lymph nodes, and lungs (16, 29). However, one of the limitations of FDG PET/CT for its utility to assess systemic disease activity is due to its limited spatial resolution, other manifestations like neuropathies, cutaneous vasculitis, and other skin abnormalities cannot be effectively evaluated.

 In the literature, the utility of FDG PET in high-grade lymphoma is well established for the site of biopsy, initial staging, response assessment to therapy, and detection of disease recurrence, and in the case of low-grade lymphoma except for initial staging, other indications hold strong for PET/CT (17-19). In the case of primary SS, several complications lead to abnormal FDG uptake like interstitial lung disease and unidentified lymphadenopathy. Involvement of the lymph nodes and parotid glands is common in the case of lymphoma, however, it is observed with similar frequency in patients without lymphoma too. One of the studies reported some specific findings that are associated with lymphoma in ^18^F-FDG–PET-CT in SS patients, particularly SUV_max_ at any site of ≥5.6, a SUV_max_ in the parotid glands of ≥4.7, and focal or nodular hypermetabolic lung lesions. Focal or nodular hypermetabolic lung lesions were exclusively observed in patients with lymphoma (26). ^18^F-FDG PET/CT may be utilized in high-risk SS patients to assess the development of lymphoma (lymphomagenesis) (30, 26). Our patient also showed up with swelling of the eyes and parotid gland and on PET/CT the patient showed an elevated SUV_max_ of 7.9 on parotid glands.

 The metabolic tracer uptake of FDG PET/CT in the most commonly seen histological type of SS patients i.e, MALT lymphoma is highly variable and mostly changes with the site of involvement. This leads to defining the role of FDG PET/CT as controversial in MALT lymphomas. Usually, SS-associated MALT lymphomas develop commonly in the salivary glands and/or lungs. Pulmonary and head/neck MALT lymphomas show the most FDG avidity with higher sensitivities to detect lesions in these regions (31, 32). In the second common histologic type, DLBCL, the role of FDG PET/CT is well established. In this scenario, there may be a role for FDG PET/CT in the detection of lymphomas associated with SS. The major advantage of FDG PET/CT in SS-associated lymphomas is its ability to detect extra-glandular involvement. As demonstrated by Kerean et al, it is also useful to guide the site of biopsy and also for response assessment evaluations (26). However benign lesions with abnormal FDG uptake should be cautiously reported as they can be misleading.

 Recently ^68^Ga-Pentaxifor, a radiolabeled chemokine receptor CXCR4, was used to assess disease activity in a SS patient. The rationale behind the intense uptake seen is due to the inflammatory cell infiltration as played by the specific receptor in the migration of inflammatory cells (33). Some studies we have also noticed overexpression of somatostatin receptors in salivary glands and joints of SS patients, as depicted by ^99m^Tc-HYNIC-TOC scintigraphy and ^68^Ga-DOTATOC PET/CT (34). 

 Furthermore, the potential role of B cell lymphocyte imaging namely ^89^Zr-rituximab in SS for whole-body visualization of B cells in various organs has been hypothesized. There is particular interest in assessing its role in treatment response post rituximab therapy. 

 Even T cell lymphocyte visualization observed during the early phase of SS is also speculated using radiolabeled interleukin-2 that is overexpressed in activated T cell lymphocytes.

## Conclusion

 In conclusion, the utility of FDG PET/CT to assess systemic disease activity can be appraised with consideration of its limitations. 

 In addition, there is a promising role of FDG PET/CT in SS-associated lymphomas. The current case report shows that FDG PET/CT may have a role in the diagnostic process of SS-associated lymphomas. Nonetheless, FDG PET/CT is useful in the systemic staging of SS-associated lymphomas, and the evaluation of treatment response. Larger cohort studies are warranted to establish its further presumed role in SS-associated lymphomas. Furthermore, new SS-specific PET tracers should be explored as they may provide promising and important insights into the pathological processes of SS. 

## References

[1] Mariette X, Criswell LA (2018). Primary Sjögren’s syndrome. N Engl J Med.

[2] Greenspan JS, Daniels TE, Talal N, Sylvester RA (1974). The histopathology of Sjögren’s syndrome in labial salivary gland biopsies. Oral Surg Oral Med Oral Pathol.

[3] Ramos-Casals M, Brito-Zerón P, Sisó-Almirall A, Bosch X (2012). Primary Sjögren syndrome. BMJ.

[4] Brito-Zerón P, Theander E, Baldini C, Seror R, Retamozo S, Quartuccio L (2016). Early diagnosis of primary Sjögren’s syndrome: EULAR-SS task force clinical recommendations. Expert Rev Clin Immunol.

[5] Signore A, Anzola K L, Auletta S, Varani M, Petitti A, Pacilio M (2018). Current status of molecular imaging in inflammatory and autoimmune disorders. Curr Pharm Des.

[6] Garcia-Carrasco M, Ramos-Casals M, Rosas J, Pallares L, Calvo-Alen J, Cervera R (2002). Primary Sjögren syndrome: clinical and immunologic disease patterns in a cohort of 400 patients. Medicine (Baltimore).

[7] Goules AV, Tzioufas AG (2019). Lymphomagenesis in Sjögren’s syndrome: Predictive biomarkers towards precision medicine. Autoimmun Rev.

[8] Stefanski AL, Tomiak C, Pleyer U, Dietrich T, Burmester GR, Dörner T (2017). The Diagnosis and Treatment of Sjögren's Syndrome. Dtsch Arztebl Int.

[9] Kassan SS, Thomas TL, Moutsopoulos HM, Hoover R, Kimberly RP, Budman DR (1978). Increased risk of lymphoma in sicca syndrome. Ann Intern Med.

[10] Pariente D, Anaya JM, Combe B, Jorgensen C, Emberger JM, Rossi JF (1992). Non-Hodgkin’s lymphoma associated with primary Sjögren’s syndrome. Eur J Med.

[11] Tzioufas AG, Voulgarelis M (2007). Update on Sjögren’s syndrome autoimmune epithelitis: from classification to increased neoplasias. Best Pract Res Clin Rheumatol.

[12] Routsias JG, Goules JD, Charalampakis G, Tzima S, Papageorgiou A, Voulgarelis M (2013). Malignant lymphoma in primary Sjögren’s syndrome: An update on the pathogenesis and treatment. Semin Arthritis Rheum.

[13] Royer B, Cazals-Hatem D, Sibilia J, Agbalika F, Cayuela JM, Soussi T (1997). Lymphomas in patients with Sjogren’s syndrome are marginal zone B-cell neoplasms, arise in diverse extranodal and nodal sites, and are not associated with viruses. Blood.

[14] Voulgarelis M, Dafni UG, Isenberg DA, Moutsopoulos HM (1999). Malignant lymphoma in primary Sjögren’s syndrome: A multicenter, retrospective, clinical study by the European concerted action on Sjögren’s syndrome. Arthritis Rheum.

[15] Seror R, Bowman SJ, Brito-Zeron P, Theander E, Bootsma H, Tzioufas A (2015). EULAR Sjögren's syndrome disease activity index (ESSDAI): a user guide. RMD Open.

[16] Cohen C, Mekinian A, Uzunhan Y, Fauchais AL, Dhote R, Pop G (2013). 18F-fluorodeoxyglucose positron emission tomography/computer tomography as an objective tool for assessing disease activity in Sjögren's syndrome. Autoimmun Rev.

[17] Jadvar H, Bonyadlou S, Iagaru A, Colletti PM (2005). FDG PET-CT demonstration of Sjogren’s sialoadenitis. Clin Nucl Med.

[18] Kumar P, Jaco MJ, Pandit AG, Shanmughanandan K, Jain A, Rajeev Ravina M (2013). Miliary sarcoidosis with secondary Sjogren's syndrome. J Assoc Physicians India.

[19] Sharma P, Chatterjee P (2015). 18F-FDG PET/CT in multisystem Sjögren Syndrome. Clin Nucl Med.

[20] Serizawa I, Inubushi M, Kanegae K, Morita K, Inoue T, Shiga T (2007). Lymphadenopathy due to amyloidosis secondary to Sjögren syndrome and systemic lupus erythematosus detected by F-18 FDG PET. Clin Nucl Med.

[21] Ma D, Lu H, Qu Y, Wang S, Ying Y, Xiao W (2015). Primary Sjögren’s syndrome accompanied by pleural effusion: A case report and literature review. Int J Clin Exp Pathol.

[22] Nakamoto Y, Tatsumi M, Hammoud D, Cohade C, Osman MM, Wahl R L (2005). Normal FDG distribution patterns in the head and neck: PET/CT evaluation. Radiology.

[23] Zincirkeser S, Sahin E, Halac M, Sager S (2007). Standardized uptake values of normal organs on 18F-fluorodeoxyglucose positron emission tomography and computed tomography imaging. J Int Med Res.

[24] Carter KR, Kotlyarov E (2007). Common causes of false positive F18 FDG PET/CT scans in oncology. Braz Arch Biol Technol.

[25] Nicolau J, Sassaki KT (1976). Metabolism of carbohydrate in the major salivary glands of rats. Arch Oral Biol.

[26] Weiler-Sagie M, Bushelev O, Epelbaum R, Dann EJ, Haim N, Avivi I (2010). 18F-FDG avidity in lymphoma readdressed: a study of 766 patients. J Nucl Med.

[27] Ghielmini M, Vitolo U, Kimby E, Montoto S, Walewski J, Pfreundschuh M (2013). ESMO guidelines consensus conference on malignant lymphoma 2011 part 1: diffuse large B-cell lymphoma (DLBCL), follicular lymphoma (FL) and chronic lymphocytic leukemia (CLL). Ann Oncol.

[28] Dreyling M, Thieblemont C, Gallamini A, Arcaini L, Campo E, Hermine O (2013). ESMO consensus conferences: guidelines on malignant lymphoma Part 2: marginal zone lymphoma, mantle cell lymphoma, peripheral T-cell lymphoma. Ann Oncol.

[29] Keraen J, Blanc E, Besson FL, Leguern V, Meyer C, Henry J (2019). Usefulness of 18F-Labeled Fluorodeoxyglucose-Positron Emission Tomography for the Diagnosis of Lymphoma in Primary Sjögren's Syndrome. Arthritis Rheumatol.

[30] Alunno A, Leone MC, Giacomelli R, Gerli R, Carubbi F (2018). Lymphoma and Lymphomagenesis in Primary Sjögren's Syndrome. Front Med (Lausanne).

[31] Perry C, Herishanu Y, Metzer U, Bairey O, Ruchlemer R, Trejo L (2007). Diagnostic accuracy of PET/CT in patients with extranodal marginal zone MALT lymphoma. Eur J Haematol.

[32] Albano D, Durmo R, Treglia G, Giubbini R, Bertagna F (2020). 18F-FDG PET/CT or PET Role in MALT Lymphoma: An Open Issue not Yet Solved-A Critical Review. Clin Lymphoma Myeloma Leuk.

[33] Cytawa W, Kircher S, Schirbel A, Shirai T, Fukushima K, Buck AK (2018). Chemokine Receptor 4 Expression in Primary Sjögren’s Syndrome. Clin Nucl Med.

[34] Anzola LK, Rivera JN, Dierckx RA, Lauri C, Valabrega S, Galli F (2019). Value of Somatostatin Receptor Scintigraphy with 99mTc-HYNIC-TOC in Patients with Primary Sjögren Syndrome. J Clin Med.

